# Importance of physical health and health-behaviors in adolescence for risk of dropout from secondary education in young adulthood: an 8-year prospective study

**DOI:** 10.1186/s12939-015-0272-x

**Published:** 2015-11-24

**Authors:** Erla Svansdottir, Sigurbjorn A. Arngrimsson, Thorarinn Sveinsson, Erlingur Johannsson

**Affiliations:** School of Education, University of Iceland, Stakkahlíð, 105 Reykjavík, Iceland; Center for Sport and Health Sciences, University of Iceland, Lindarbraut 4, 840 Laugarvatn, Iceland; Research Centre of Movement Science, School of Health Sciences, University of Iceland, Stapi v/Hringbraut, 101 Reykjavík, Iceland; Landspitali-University Hospital, Eiríksgötu 5, 101, Reykjavík, Iceland

**Keywords:** Adolescence, Health Inequalities, Education, Gender, Health-behaviors

## Abstract

**Background:**

Education and health constitute two interlinked assets that are highly important to individuals. In Iceland, prevalence of dropout from secondary education poses a considerable problem. This 8-year prospective study assesses to what extent poor physical health and negative health-behaviors of Icelandic adolescents predict increased odds of dropout from secondary education.

**Methods:**

The sample included *n* = 201 Icelandic children who participated at age 15 (baseline) and again at age 23 (follow-up). Data included objective measurements of physical health and questionnaires assessing health-behaviors, education status, parental education, neighborhood characteristics, self-esteem, and depression. Independent t-tests and chi-square were used to assess differences in physical health and health-behaviors at follow-up stratified by education status. Ordinal regression models were conducted to assess whether physical health and health-behaviors at age 15 predicted increased odds of dropout from secondary education at age 23, independent of gender, parental education and psychological factors.

**Results:**

At age 23, 78 % of girls and 71 % of boys had completed a secondary education. Completion of a secondary education was associated with significant health benefits, especially among women. Women without a secondary education had lower fitness, more somatic complaints, higher diastolic blood pressure, less sports participation, and poorer sleep, whilst men without a secondary education watched more television. In logistic regression models somatic complaints during adolescence were associated with 1.09 (95 % CI: 1.02-1.18) higher odds of dropout from secondary education in young adulthood, independent of covariates. Health-behaviors associated with higher dropout odds included smoking (3.67, 95 % CI: 1.50-9.00), alcohol drinking (2.57, 95 % CI: 1.15-5.75), and time spent watching television (1.27, 95 % CI:1.03-1.56), which were independent of most covariates. Finally, mother’s higher education was strongly associated with significantly lower dropout odds (OR 0.54, 95 % CI: 0.34-0.88) independent of father’s education and psychological factors, whilst high self-esteem was independently associated with lower dropout odds (OR 0.91, 95 % CI: 0.85-0.98).

**Conclusions:**

Completion of a secondary education yields substantial physical health benefits for young women, but not for men. Importantly, somatic complaints and negative health-behaviors among adolescent boys and girls adversely impact their educational outcomes later in life, and may have widespread consequences for their future prospects.

## Background

Education is an important asset for individuals in today’s society. Completion of secondary education provides young adults with the technical and vocational skills needed to secure good jobs and/or the rights to progress to university [1]. Hence, not completing a secondary education can lead to considerable costs to individuals, through lower earnings or unemployment, and the society, via lower tax revenue and higher spending on public assistance [2].

Education is considered of high importance in Iceland, as all children and young adults are given an equal right to education, free of charge, in both compulsory and upper secondary school. In the Icelandic Education System students attend compulsory school from ages 6–15, whilst secondary education is typically planned from ages 16–19 [3]. However, dropout from secondary education in the Icelandic setting is a considerable problem, with a prevalence of 30 %, and only 45 % of students graduate on time [4]. In light of this, the Ministry of Education, Science and Culture has put forward the goal to increase completion of secondary education in Iceland on time to 60 % by 2018 [5].

One way to prevent dropout from secondary education could be through health promotion, focusing on students’ physical and mental health within secondary schools. An inquiry made by the Ministry of Education, Science and Culture among Icelandic secondary schools in 2013 has noted that 17 % of dropout students cited health-related reasons for their dropout, including physical and mental health problems (with other main reasons comprising for example failed attendance (25 %), started working (11 %), transferred to another school (12 %), lack of interest (4 %), and financial difficulties (3 %) [6]. Large longitudinal studies have likewise linked low general health status, obesity, and health problems with higher odds of dropout from secondary education [7, 8], indicating that poor health may be an influential factor for lower educational attainment among youths.

Importantly, a report from the World Health Organization (WHO) has specifically criticized how little emphasis western countries have given the influence of health and health-behaviors of children and adolescents on their educational attainment. In the report they question the one directionality in research on this topic, that is why the vast majority of studies have only investigated how lower educational attainment is associated with poorer health (i.e. the educational gradient in health [9]), but not how health factors may affect educational attainment [10]. Notably, very few studies have investigated this topic in Europe [11], and the ones that have are moreover limited to a certain extent as they have mostly been based on infant or self-report health data [12], or short-term academic outcomes [13]. Data regarding how psychological factors affect this association are also scarce, particularly concerning depression [10]. Lastly, few studies have adjusted for the possible confounding effects of parental education, an important index of socioeconomic status [13, 14], and neighborhood characteristics. Although Iceland contains a largely homogeneous population, with a low unemployment rate and an education and healthcare system similar to the other Nordic countries [15] there is some evidence that socio-economic and neighborhood differences in health and well-being are present within the country. Recent findings have for instance reported that socioeconomic inequalities are largest in Iceland of all the Nordic countries [16], and linked certain neighborhood characteristics with more substance abuse among adolescents [17]. Importantly, these factors might influence risk of dropout from secondary school as well, which would directly conflict with the fundamental principle of the Icelandic Education System concerning equal rights for acquiring an education regardless of gender, social status, cultural background, or residential location [18].

Thus, the objective of the current study is to investigate prospectively the relationship between health and education among Icelandic adolescents, with a specific focus on how physical health and health-behaviors during adolescence influence educational attainment in young adulthood. More specifically, the aims are: 1) to evaluate differences in the physical health, health-behaviors, and psychological factors of young adults who have or have not completed a secondary education; 2) to examine how physical health and negative health-behaviors of adolescents affect their odds of dropout from secondary education; and 3) to examine if such associations remain after controlling for parental education and psychological factors. We hypothesize that young adults who have completed a secondary education will report better physical health, more positive health-behaviors, and a better standing on psychological factors, and that poorer standing on physical health and health-behaviors will be linked with a higher risk of dropout from secondary education, independent of covariates.

## Methods

### Participants

**Fig. 1:**
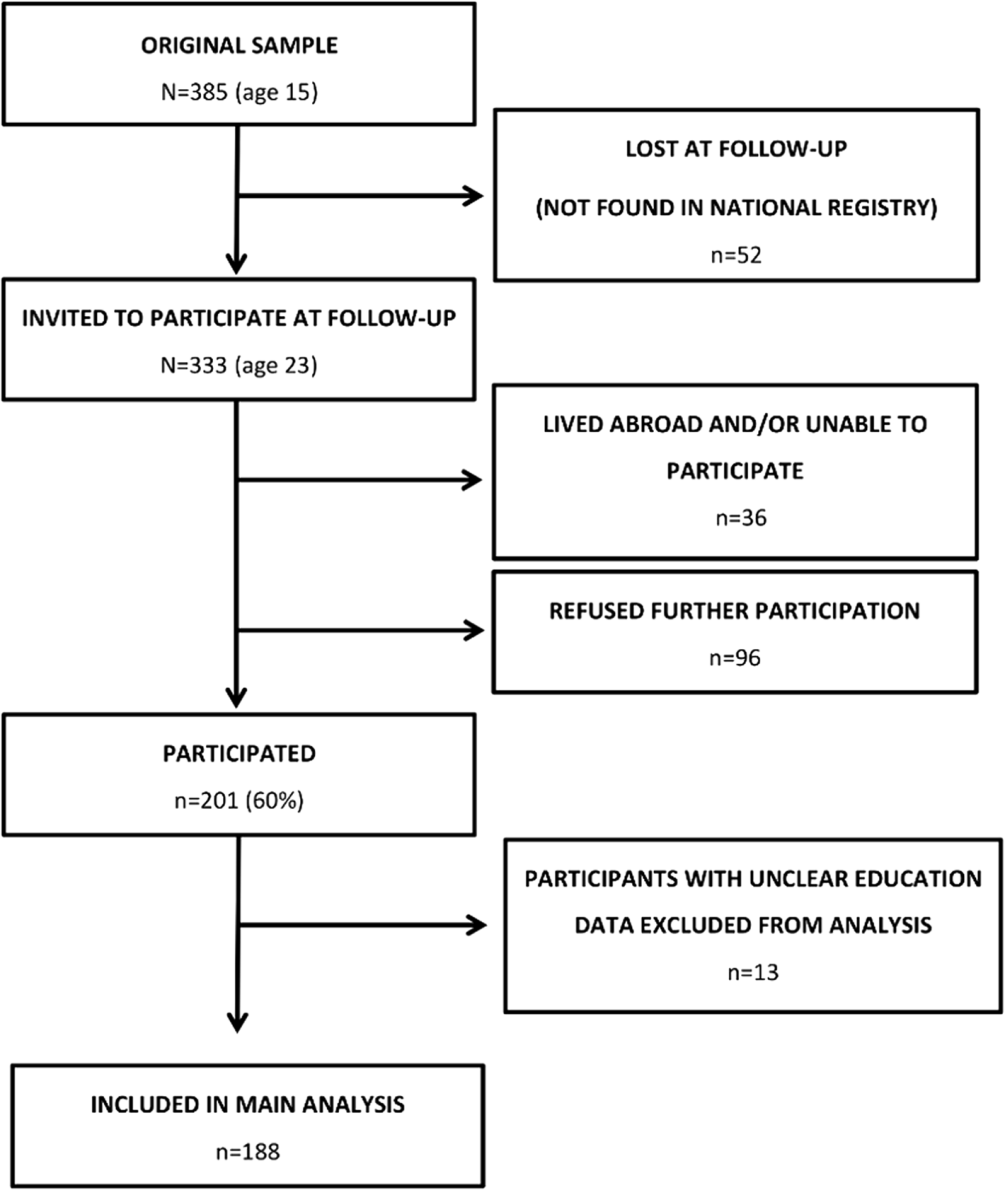
Overview of participation and dropout from the original and follow-up study

This study is based on a previous sample of *N* = 385 children (195 boys, 190 girls) born in 1988 from 18 schools selected based on the geographical distribution in Iceland, with 60 % of participants from the metropolitan Reykjavik-area and 40 % from the north-east of Iceland. This sample constitutes 10 % of the overall population of Icelandic citizens at this age group. These students were originally approached for participation in 2003 when they attended 10th grade (age 15) of primary school, and a written informed consent was obtained from parents and participants. For the current study, a follow-up was conducted eight years later when participants were 23-years old. At the follow - up, *n* = 333 (86 %) of the original sample was successfully located and invited to partake in the study. The *n* = 52 individuals lost at follow - up were subjects who were not found in the National Registry and had most likely moved abroad with their parents or for studies. The scheme for participation in the follow-up study is displayed in detail in Fig. 1. The Icelandic Bioethics Committee approved the study and it was conducted according to the ethical tenets of the Declaration of Helsinki. The study design and measurements were conducted in line with the protocol of the European Youth Heart Study [19].

### Measures

#### Demographics: socio-economic status and neighborhood characteristics

Information regarding parental education (what level of education participants’ mothers and fathers had completed (i.e. *primary education, vocational education, matriculation examination, university degree*)) was collected from participants at follow-up, and used as a proxy measure for socio- economic status. Participant’s occupation status and child-rearing responsibilities (whether they had children) were assessed with self-report at follow-up. Neighborhood characteristics of participants were defined by the local-area their respective schools resided in. These were categorized into two areas: a) metropolis (Reykjavik capital area (six schools), and urban/rural (12 schools from smaller towns and rural areas).

### Physical health

Objective measures of physical health were conducted at both baseline and follow-up. Of note, due to limited funding resources at baseline, objective measures of fitness (with a bicycle ergometer) and physical activity (with accelerometers) were only conducted among half of the participant sample at baseline. Thus, half of participants from each student class were invited to undergo fitness and physical activity measures at baseline. Conversely, these two measures were conducted among all participants at follow-up. Fitness was assessed with performance on a maximal graded bicycle ergometer (Monark 839E) test [20, 21]. A description of the test was given, and the importance of maximal effort was stressed, but participants were also told they could stop cycling at any time. The test has been validated in adolescents [20]. Physical activity was objectively measured with Actigraph activity monitors (model GT3X). Participants carried activity monitors secured by waist belt at their right hip for six consecutive days including both weekend days. These monitors have been validated in youths [22, 23], and the measurement used was counts per minute. Standing height was measured with a transportable stadiometer to the nearest millimeter, and body weight was determined to the nearest 0.1 kg on a scale. Body Mass Index (BMI) was calculated as body mass (kg) divided by height (m) squared. Waist-circumference and skinfold thickness were assessed using standardized procedures. Blood pressure was measured at follow-up using a validated, semi-automated device (ADC Advantage) after participants had rested for ten minutes. Measures were taken over the brachial artery three times. The average of the last two recordings was used. The Somatization subscale of the Symptom Checklist 90 (SCL-90) [24] was used to assess occurrences of subjective and psychological health complaints (e.g. stomach ache and headache) in the past seven days. The subscale contains eight items scored on a five-point Likert scale.

### Health - behaviors

Health-behavior measures included the following at baseline and follow-up: a) smoking in the past 30 days (*no, yes*); b) alcohol consumption in the past 30 days (*no, yes*); c) number of hours spent watching television each weekday (*almost never, 30–60 min, ≈1 h, ≈2 h, ≈3 h, ≈4 h, ≈5 h, ≥6 h*); and d) how often participants engaged in moderate/vigorous physical activity (d.1) or sports (d.2) (*never/rarely, 1–3 times a week, ≥4 times a week*). The following health-behaviors were only assessed at follow-up: a) “How many hours do you spend sleeping on weekdays?” *(≤7 h per night, ≥8 h*); and b) “How often do you get enough sleep?” (*very seldom, about half of the time, most nights, sleep too much*). Prior to analyses, categories for b) were combined into a binary variable indicating “I don’t get enough sleep” (*very seldom/half of the time*) versus “I get enough sleep” (*most nights/sleep too much)*.

### Psychological factors

Measures of psychological factors included self-esteem and depression, which were measured at baseline and follow-up, and life satisfaction and social support, which were assessed at follow-up only. Self-esteem was assessed using the Rosenberg Self - Esteem Scale [25]. The scale includes ten statements with four response options. Total scores range from 0–30 with higher scores reflecting greater self-esteem. The psychometric properties of the Icelandic version have been deemed sufficient [26]. The Depression subscale of the SCL-90 [24] was used to assess symptoms of depression in the preceding week. The ten-item subscale is scored on a five-point Likert scale asking about feelings the last seven days. Life satisfaction was assessed with Dieners’ Satisfaction with Life Scale [27], a five-item scale measuring global cognitive judgment of one’s life satisfaction. Participants indicate on a seven-point scale how much they agree or disagree with each item. Finally, the Multidimensional Scale of Perceived Social Support [28] was used to measure social support. Psychometric evaluations have verified the reliability and validity of the scale among adolescents [29].

### Education completion

Participants reported at follow-up what education they had completed (i.e. *primary education*, *vocational education*, *matriculation examination*, *university education*). In the Icelandic education system students usually complete elementary school at age 15–16 and vocational/matriculation examination at age 19–20 [3]. Dropout from secondary education was defined by only having an elementary education by age 23, whilst having completed a vocational-, matriculation-, and/or university education was categorized as “*has completed a secondary education*”. Participants with “other education” were excluded from analyses due to lack of information regarding their education (*n* = 7 girls and *n* = 6 boys). No differences were noted in living location, gender, parental education status, nor in any measure of physical health at baseline or follow-up between participants and excluded subjects, except that those excluded from analyses had lower BMI at baseline (M = 20.9 (SD 2.9) vs. M = 19.1 (SD 1.6), p = 0.029) and follow-up (M = 24.4 (SD 3.9) vs. 21.7 (SD 2.8), *p* = 0.013), and a lower waist-circumference (M = 78.5 cm (SD 10.8) vs. M = 71.7 cm (SD 8.4), *p* = 0.025) at follow-up.

### Statistical analyses

Independent t-tests, chi-square, and tau-c rank correlations were used to a) assess gender differences in demographics and covariates at baseline (i.e. parental education, neighborhood characteristics, and psychological factors); and b) examine differences in physical health measures, health-behaviors and socio-demographics at follow-up by educational status, across the whole sample and stratified by gender (i.e. among girls and boys separately).

A logistic regression was conducted to assess the influence of physical health, health-behaviors and covariates at baseline on odds of dropout from secondary education. Three different hierarchal models were executed in the whole sample, using the stepwise method and controlling for different covariates per model. In all models a single independent variable (one physical health or health-behavior measure) was inserted at the 1st step and gender at the 2nd step. The models differed however at the 3rd step, with Model 1 containing no further adjustment, while Model 2 was adjusted for mother’s and father’s education status and neighborhood characteristics; and Model 3 adjusted for baseline self-esteem and depression. These models were subsequently repeated for each covariate (i.e. gender, mother’s higher education, father’s higher education, neighborhood characteristics, self-esteem, and depression) to assess their independent effects. In these models, each covariate was inserted at the 1st step, with steps 2–3 run as described above except that the relevant covariate was excluded from later steps.

## Results

Demographics are presented in Table 1. Participants education status differed by gender (Tau-c (*N* = 201) = −0.19, *p* = 0.01). More boys had completed vocational studies (*χ*^2^ (1,201) = 9.333, *p* = 0.002), while more girls had completed a matriculation examination (*χ*^2^ (1,201) = 9.158, *p* = 0.002). Using the aforementioned definition of secondary education, 78 % of girls (*n* = 72) and 71 % of boys (*n* = 78) had completed a secondary education by age 23.

**Table 1:** Demographics and covariates at baseline for the whole sample and across gender

Demographics and covariates at baseline	Whole sample *N* = 201	Girls *N* = 92	Boys *N* = 109	*p*-value
Age (baseline) M (SD)	15.3 (0.3)	15.3 (0.3)	15.4 (0.3)	0.017
Education of participants				
Elementary education	19 % (38)	14 % (13)	23 % (25)	0.11
Vocational studies	13 % (27)	5 % (5)	20 % (22)	0.002
Matriculation examination	49 % (99)	61 % (56)	39 % (43)	0.002
University degree	12 % (24)	12 % (11)	12 % (13)	1.00
Other	7 % (13)	8 % (7)	6 % (6)	0.55
Mother’s education status				
Elementary education	23 % (45)	21 % (19)	24 % (26)	
Secondary education	37 % (74)	38 % (35)	36 % (39)	0.86^*^
University degree	40 % (80)	41 % (37)	40 % (43)	
Father’s education status				
Elementary education	19 % (37)	20 % (18)	18 % (19)	0.74^*^
Secondary education	51 % (101)	50 % (45)	52 % (56)	
University degree	30 % (61)	30 % (28)	30 % (30)	
Neighborhood characteristics				
Capital area	50 % (100)	50 % (46)	49 % (54)	0.95
Urban/ Rural	50 % (101)	50 % (46)	51 % (55)	
Psychological factors				
Self-esteem (baseline) M (SD)	21.7 (5.6)	21.0 (5.7)	22.3 (5.5)	0.12
Depression (baseline) M (SD)	15.5 (6.0)	16.8 (7.2)	14.4 (4.6)	0.008

Numbers in the table represent percentage (number of participants) unless other specified

*M* mean, *SD* standard deviation

^*^Significance estimated with rank (tau-c) correlation coefficient

### Health differences in young adulthood by education status

Differences in physical health, health-behaviors, and psychological factors at follow-up by education status are presented in Table 2. Across the whole sample, individuals who had completed a secondary education reported higher life satisfaction, less somatic complaints, less television watching, and more sleep. No differences were found in physical health measures. In gender stratified analyses differences in life satisfaction by education remained among both genders, but specific gender differences emerged for physical health measures. That is, women without a secondary education had lower fitness, more somatic complaints and higher diastolic blood pressure, as well as marginally higher waist-circumference compared with women with a secondary education. No differences were seen in men, aside from a trend towards more somatic complaints (*p* = 0.07). Regarding health-behaviors, men without a secondary education reported more time spent watching television, while women without a secondary education reported less sports participation and worse sleep. Notably, 70 % of women with elementary education never/rarely exercised, compared with 35 % of women with a secondary education (*p* = 0.023). Moreover, 46 % of women with elementary education versus 5 % of women with a secondary education reported seldom getting enough sleep (*p* < 0.001), with 15 % vs. 63 % of these women (respectively) stating they slept ≥7 h each night (*p* < 0.002). Elementary educated women were also more likely to have child-raising responsibilities, as 46 % of them reported having children as compared with 13 % of women with a secondary education (*p* < 0.01). Lastly, occupational status differed by education status across the whole sample, with a higher prevalence of unemployment and working full-time among elementary educated participants, and a higher prevalence of current studying and working part-time among participants with a secondary education.

**Table 2:** Differences in physical health, health - behaviors and psychological factors at follow-up by education status

	Total sample		Girls		Boys	
	Elementary education (*n* = 38)	Secondary education (*n* = 150)	Elementary education (*n* = 13)	Secondary education (*n* = 72)	Elementary education (*n* = 25)	Secondary education (*n* = 78)
Physical health						
Fitness (W/kg)	2.7 (0.7)	2.8 (0.6)	2.0 (0.5)**	2.5 (0.5)**	2.9 (0.5)	3.0 (0.5)
Physical activity (counts/min)	559 (208)	555 (186)	492 (82)	492 (122)	581 (236)	615 (216)
Body mass index (kg/m^2^)	24.6 (4.5)	24.4 (3.8)	24.7 (4.8)	23.6 (3.3)	24.6 (4.2)	25.1 (4.0)
Waist circumference (cm)	79.4 (10.8)	78.4 (11.0)	76.8 (11.1)	72.1 (7.2)	80.8 (9.3)	84.2 (10.8)
Skinfold thickness (cm)	114.6 (58.1)	120.0 (50.3)	150.1 (70.6)	130.6 (48.3)	94.1 (37.9)	110.2 (50.4)
Somatic complaints	15.3 (7.4)**	13.1 (4.3)**	16.5 (5.6)*	13.5 (4.4)*	14.7 (4.9)	12.8 (4.1)
Systolic blood pressure (mm/Hg)	121 (11.9)	117.3 (11.4)	114.4 (10.3)	111.1 (8.6)	124.7 (11.2)	122.9 (10.8)
Diastolic blood pressure (mm/Hg)	74.0 (7.1)	72.5 (6.0)	76.6 (8.9)*	71.5 (6.6)*	72.7 (5.7)	73.3 (5.7)
Health - behaviors						
Smoking	5 % (2)	3 % (4)	8 % (1)	1 % (1)	4 % (1)	4 % (3)
Alcohol drinking	82 % (31)	86 % (127)	92 % (12)	86 % (62)	76 % (19)	87 % (65)
Moderate/vigorous physical activity						
* Never/rarely*	19 % (6)	8 % (11)	30 % (3)	9 % (6)	14 % (3)	7 % (5)
* 1-3 times a week*	22 % (7)	33 % (47)	30 % (3)	39 % (27)	18 % (4)	28 % (20)
* ≥4 times a week*	59 % (19)	59 % (83)	40 % (4)	52 % (36)	68 % (15)	65 % (47)
Physical activity with a sports club						
* Never/rarely*	40 % (13)	31 % (44)	70 % (7)*	35 % (24)*	27 % (6)	28 % (20)
* 1-3 times a week*	13 % (4)	23 % (33)	20 % (2)*	26 % (18)*	9 % (2)	21 % (15)
* ≥4 times a week*	47 % (15)	45 % (64)	10 % (1)*	39 % (27)*	64 % (14)	51 % (37)
Watch television >2 h on weeknights	47 % (18)*	27 % (40)*	39 % (5)	26 % (19)	52 % (13)*	27 % (21)*
Seldom get enough sleep	27 % (9)**	6 % (9)**	46 % (6)**	5 % (4)**	12 % (3)	6 % (5)
Sleep ≥7 h each night	32 % (12)**	55 % (83)**	15 % (2)**	63 % (45)**	40 % (10)	49 % (38)
Psychological factors						
Self-esteem	21.5 (6.4)	23.4 (5.4)	21.5 (6.1)	23.9 (5.0)	21.6 (6.6)	22.9 (5.8)
Depression	16.8 (7.4)	14.8 (4.9)	18.2 (7.9)	15.4 (5.5)	16.0 (7.1)	14.2 (4.3)
Life satisfaction	22.8 (6.5)**	26.3 (4.9)**	23.5 (4.8)*	27.1 (4.8)*	21.3 (7.3)*	25.5 (4.8)*
Social support	17.3 (2.9)	18.1 (2.3)	36.4 (4.4)	36.3 (3.6)	31.8 (5.7)	33.7 (4.6)
Demographics						
Child-rearing responsibilities	26 % (10)	14 % (21)	46 % (6)**	13 % (9)**	16 % (4)	15 % (12)
Working full-time	47 % (18)**	21 % (32)**	23 % (3)	17 % (12)	60 % (15)**	26 % (20)**
Unemployed	13 % (5)**	1 % (2)**	15 % (2)*	1 % (1)*	12 % (3)*	1 % (1)*
Working part-time	18 % (7)**	41 % (62)**	23 % (3)	47 % (34)	16 % (4)	36 % (28)
Studying	42 % (16)**	76 % (114)**	46 % (6)**	82 % (59)**	40 % (10)**	71 % (55)**

Numbers in the table represent: Mean (Standard Deviation) for continuous variables (all Physical health and Psychological factors variables) and % (number of participants) for categorical variables (all Health -behavior and Demographic variables)

*p < 0.05; **p < 0.01

### Influence of adolescents’ physical health and health-behaviors on dropout odds

In logistic regression analyses across the sample (see Table 3) somatic complaints, smoking, alcohol drinking and time spent watching television were associated with higher odds of dropout, while self-esteem and mothers’ education were associated with lower odds of dropout from secondary education. No association emerged however between objective physical health measures, consisting of fitness, physical activity and body composition measures, and odds of dropout.

**Table 3:** Association of physical health, health-behaviors and covariates with dropout odds from secondary education

	Model 1^a^		Model 2^b^		Model 3^c^	
	Adjusted for gender		Adjusted for gender, parental education and neighborhood characteristics		Adjusted for gender and psychological factors	
Baseline measures	*N*	OR (95 % CI)	*N*	OR (95 % CI)	*N*	OR (95 % CI)
Physical health						
Fitness (W/kg)	114	0.68 (0.33-1.41)	110	0.55 (0.25-1.19)	107	0.84 (0.34-2.09)
Physical activity (counts/min)	80	1.00 (1.00-1.00)	77	1.00 (0.99-1.00)	76	1.00 (1.00-1.00)
Body mass index (kg/m2)	188	1.05 (0.93-1.18)	184	1.07 (0.94-1.20)	172	1.10 (0.94-1.27)
Waist circumference (cm)	188	1.02 (0.98-1.06)	184	1.03 (0.98-1.07)	172	1.03 (0.97-1.08)
Skinfold thickness (mm)	188	1.01 (0.99-1.02)	184	1.01 (0.99-1.02)	172	1.00 (0.99-1.02)
Somatic complaints	170	1.09 (1.02-1.18)*	167	1.09 (1.01-1.18)*	168	1.10 (1.00-1.20)*
Health - behaviors						
Smoking	173	3.67 (1.50-9.00)**	170	3.64 (1.42-9.33)**	171	2.90 (1.14-7.40)*
Alcohol drinking	163	2.57 (1.15-5.75)*	160	2.14 (0.93-4.93)	161	2.36 (1.03-5.40)*
Time spent watching television	173	1.27 (1.03-1.56)*	170	1.28 (1.03-1.60)*	171	1.25 (1.01-1.55)*
Frequency of sports participation	173	0.84 (0.54-1.30)	170	0.82 (0.52-1.30)	171	0.94 (0.59-1.49)
Frequency of moderate/vigorous physical activity	173	0.74 (0.42-1.92)	170	0.81 (0.46-1.45)	171	0.87 (0.49-1.57)
Covariates						
Gender (female)	188	1.78 (0.85-3.73)	184	1.51 (0.70-3.27)	172	2.42 (1.02-5.75)*
Mothers higher education	186	0.54 (0.34-0.88)*	184	0.57 (0.33-0.98)*^d^	170	0.53 (0.31-0.91)*
Fathers higher education	186	0.70 (0.42-1.19)	184	0.86 (0.47-1.58)^e^	171	0.78 (0.43-1.39)
Neighborhood characteristics (rural/urban)	188	1.10 (0.54-2.26)	184	0.80 (0.37-1.74)^f^	172	1.15 (0.52-2.56)
Self-esteem	174	0.91 (0.85-0.98)*	171	0.90 (0.84-0.97)**	172	0.92 (0.85-0.99)*^g^
Depression	172	1.06 (0.99-1.13)	169	1.06 (1.00-1.14)	172	1.02 (0.95-1.10)^h^

*p < 0.05; **p < 0.01. ^a^Model adjusted for gender; ^b^Model adjusted for gender, parental education and neighborhood characteristics ; ^c^Model adjusted for gender, self-esteem and depression; ^d^Only adjusted for fathers education status and neighborhood characteristics; ^e^Only adjusted for mothers education status and neighborhood characteristics; ^f^Only adjusted for mothers and fathers education status; ^g^Only adjusted for depression at age 15; ^h^Only adjusted for self-esteem at age 15

In total, three regression models (see Table 3) were conducted which included adjustments for possible confounders (i.e. gender in model 1, parental education and neighborhood characteristics in model 2, and psychological factors (self-esteem and depression) in model 3). In these analyses, somatic complaints remained a significant predictor associated with higher odds of dropout across all models (with OR 1.09, 95 % CI:1.02-1.18 in model 1, and nearly identical ORs in models 2 and 3). In the realm of health-behaviors, smoking at age 15 was associated with fourfold higher odds of dropout from secondary education, independent of gender (OR 3.67, 95 % CI:1.50-9.00), or parental education and neighborhood characteristics (OR 3.64, 95 % CI:1.42-9.33), and threefold odds of dropout after adjustment for psychological factors (OR 2.90, 95 % CI:1.14-7.40). Alcohol drinking was likewise associated with approximately 2.5 higher odds of dropout, independent of gender (2.57, 95 % CI:1.15-5.75) and psychological factors (2.36, 95 % CI:1.03-5.40), but the association lost significance when parental education status and neighborhood characteristics was taken into account. Time spent watching television also emerged as an independent predictor, where a one unit increase in television watching was linked with 1.3 higher odds of dropout in young adulthood across all models (e.g. OR 1.27, 95 % CI:1.03-1.56 in Model 1).

Among covariates, self-esteem was associated with lower odds of dropout at age 23, independent of other covariates in models 1–3 (e.g. OR 0.91, 95 % CI: 0.85-0.98 for Model 1). Finally, mother’s higher education status at follow-up was also strongly associated with lower dropout odds, independent of gender (OR 0.54, 95 % CI: 0.34-0.88), father’s education and neighborhood characteristics (OR 0.57, 95 % CI: 0.33-0.98), and psychological factors (OR 0.53, 95 % CI: 0.31-0.91). No association was found however with fathers’ education and neighborhood characteristics (see Table 3).

## Discussion

This prospective study spanning eight years reports on the interrelationship between adolescent’s health and educational attainment in young adulthood, focusing on how physical health and health-behaviors of youths affect their odds of dropout from secondary education. A completion of secondary education in young adulthood was associated with less somatic complaints, more sports participation, less television watching, poorer sleep, higher life satisfaction, and occupational status. This is consistent with the well-established educational/social gradient in health, where lower education has consistently been associated with poor health, including negative health-behaviors, morbidity and mortality [9]. These findings strongly suggest that an educational health gradient does exist in Iceland, but according to a report of the OECD it is only very recently that Icelandic authorities have started acknowledging the existence of health inequalities among its inhabitants [30]. Recent data from Sweden have likewise indicated a presence of social inequalities in mental health of adolescents in a country with small social differences [31]. In the current study, girls especially displayed substantial health benefits from gaining a secondary education, as compared with boys. Of specific interest were the low fitness, poorer bodily health, higher blood-pressure, lack of sleep, and more child-rearing responsibilities among women without a secondary education. Perhaps secondary education is more vital for the well-being of Icelandic women as they may be less likely than men to be able to secure a well-paying job without an educational degree. Moreover, it can be hypothesized that getting pregnant at a young age may hinder these women in completing their secondary education, and that more support is needed for young mothers in Iceland.

Regarding the prognostic importance of physical health for education outcomes, somatic complaints, negative health - behaviors, self-esteem and mothers education status emerged as significant risk factors for dropout from secondary education. Somatic complaints were linked with higher odds of dropout from secondary education, independent of both psychological factors and the socioeconomic status of students. This concurs with recent large scale studies which have linked self-reported poor health with lower odds of completing high school independent of socioeconomic status [8, 32]. No association emerged however between objective measures of physical activity or fitness with dropout, contrary to a large longitudinal study which found a relationship between academic achievement and objective measures of physical activity [33]. This may be due to lower power based on the smaller sample in the current study. Among health-behaviors, smoking and more television watching predicted higher odds of dropout from secondary education regardless of all covariates, while alcohol drinking predicted dropout independent of gender and psychological factors, but not when parental education was taken into account. According to this, parental influences may reduce the effects of adolescent drinking on lower educational attainment, but review findings have suggested that negative health-behaviors have a more complex relationship with academic achievement, and may be mediated by socioeconomic status and psychosocial problems [34]. Indeed, data from Norway, Sweden, Denmark, Finland, and the United Kingdom have found social inequalities in daily smoking among adolescents, and that this association was partially mediated by academic achievement [35].

Importantly, mother’s higher education emerged as a protective factor against dropout, but no association was found with father’s education or neighborhood characteristics. It is well established that parental education denotes socio-economic status and is an important predictor of children’s educational outcomes [14]. Contrary to these findings, the Icelandic Ministry of Education, Science and Culture has recently claimed that socio-economic status has a relatively little impact on the education achievement of Icelandic students [5]. A higher level of parental education is thought to contribute to better academic achievement via a more supportive home learning environment, encouragement for higher achievement and psychological support [36]. It is unclear why no association was found with fathers’ higher education, but very few studies have investigated the individual contribution of mothers and fathers on the educational outcomes of their children [37]. One American study has though marked fathers education as more important for boys advancement to higher education, as compared with girls [38]. It may be that the supporting role provided by educated mothers is more vital for the educational attainment of Icelandic students. The unexpected lack of association between neighborhood characteristics and dropout risk possibly results from low power due to the relatively low number of participants in the current study. Published numbers from the Statistics Iceland for all students starting secondary school in Iceland in the year 2004 indicate that 25 % of students living in the Reykjavik capital area dropout from secondary education within four years compared with 32 % of students living in urban or rural areas. Likewise, based on their numbers around 49 % of students in the capital area complete their secondary education within the allocated four years compared with 37 % of students living in the countryside [39]. Yet, a report from the OECD states that the socio-economic background of Icelandic students has less of an effect on their academic achievement compared to other OECD countries [4]. Hence, it seems that Icelandic authorities and further research projects need to ascertain to what extent socio-economic inequalities in health and education exist within the Icelandic society.

The strength of this study resides in the longitudinal design, objective measures of physical health, and taking into account the effects of psychological and socio-economic status. The main limitations of the current study include the presence of some participant dropout from baseline to follow-up, which may increase risk of selection bias. Of note, non-responders may represent a group with higher dropout rates from secondary education, and this possible selection bias might thus yield attenuated associations in the study findings [8]. Secondly, objective measures of fitness and physical activity were only conducted among half of the sample at baseline, which reduces statistical power for analyses including those variables. Finally, measurements of education completion were based on self-report, limiting the robustness of the measurements.

## Conclusions

This study yields important information regarding diverse health-benefits associated with obtaining a secondary education degree in a European country, and how physical health and health-behaviors of adolescents affect their likelihood of gaining such a degree. The findings suggest that somatic complaints and negative health-behaviors among adolescents increase their chances of dropout from secondary education, and that securing a secondary education degree yields numerous health-benefits, specifically among women. The potential pathways behind this association are believed to range across many health factors. Poor health in adolescents may for instance lead to lower levels of schooling by means of more days missed at school [40], and lower capability to learn in school [32]. Of specific concern in this regard are the lower levels of fitness and physical activity seen among adolescents today [41], which can result in poorer health status accompanied by more somatic complaints, headaches, and bodily discomfort, that can impede students capacity and productivity in school. In line with this are study findings which have linked migraine headaches with lower educational attainment [42] and frequent primary care visits with more days absent from school [43] and higher odds of dropout [40]. Accordingly, it seems poor physical health during adolescence can have wide - ranging consequences for the future prospects of youths [32]. Leading educational organizations and policy makers in the United States are increasingly recognizing the important role schools play for addressing health issues among youths [44], and this view should be incorporated into public health policies worldwide.

## Abbreviations

BMI: Body mass index
OECD: Organization for Economic Co-operation and Development
OR: Odds ratio
SCL-90: Symptom checklist 90
